# Supplementary material for: Accuracy of rating scales for evaluating aphasic patients’ psychological aspects and language function: A scoping review protocol
Source: PLoS One. 2023 May 16;18(5):e0281231. doi: 10.1371/journal.pone.0281231 (PMC10187900; doi:10.1371/journal.pone.0281231)
Supplement: S1 File — (DOCX) [file pone.0281231.s002.docx]

**Draft of search strategy to be used using PubMed electronic database**

| **Components** | **Search items** | **Results** |
| --- | --- | --- |
| #1 | **Stroke :** **"Stroke"[MeSH Terms] OR "stroke"[Text Word] OR "apoplexy"[Text Word] OR "hemorrhage"[Text Word] OR "poststroke"[Text Word] OR (("cerebral"[Text Word] OR "brain"[Text Word] OR "ischemic"[Text Word] OR "intracerebral"[Text Word] OR "vascular"[Text Word] OR "cerebrovascular"[Text Word] OR "cerebral vascular"[Text Word]) AND ("accident"[Text Word] OR "infarct*"[Text Word] OR "hemorrhag*"[Text Word] OR "injury"[Text Word] OR "thrombosis"[Text Word]))** | **962,760** |
| #2 | **Aphasia : "Aphasia"[MeSH Terms] OR "aphasi*"[Text Word] OR "anomi*"[Text Word] OR "alogi*"[Text Word] OR "anepi*"[Text Word] OR "dysphasi*"[Text Word] OR "word deafness"[Text Word] OR lichtheim[Text Word]** | **23,376** |
| #3 | #1 AND #2 | **7671** |
| #4 | **Rating scales:** "Neuropsychological Tests"[MeSH Terms] OR "Health Status Indicators"[MeSH Terms] OR "test*"[Title/Abstract] OR "measure*"[Title/Abstract] OR "index"[Title/Abstract] OR "screening"[Title/Abstract] OR "scale*"[Title/Abstract] OR "status"[Title/Abstract] OR "point*"[Title/Abstract] | 9653,441 |
| #5 | "Communication"[MeSH Terms] OR "communicati*"[Text Word] OR "ｌanguage"[Text Word] OR "speech"[Text Word] OR "read*"[Text Word] OR "writ*"[Text Word] OR "vocabulary"[Text Word] OR "linguistic"[Text Word] OR "discourse"[Text Word] OR "Quality of Life"[MeSH Terms] OR "quality of life"[Text Word] OR "well being"[Text Word] OR "wellbeing"[Text Word] OR "life quality"[Text Word] | 2,034,764 |
| #6 | #3 AND #4 AND #5 | 2,592 |
| #7 | #6 NOT ("Clinical Laboratory Techniques"[MeSH Terms] OR "Diagnostic Imaging"[MeSH Terms] OR "drug therapy"[MeSH Subheading]) | 1,788 |
