# Supplementary material for: Accuracy of rating scales for evaluating aphasic patients’ psychological aspects and language function: A scoping review protocol
Source: PLoS One. 2023 May 16;18(5):e0281231. doi: 10.1371/journal.pone.0281231 (PMC10187900; doi:10.1371/journal.pone.0281231)
Supplement: Attachment — Submitted filename: FinalFile_Reviewer_Comments.docx [file pone.0281231.s003.docx]

PLOS ONE_ First revision_20230331

Upon scholarly discourse amongst the authors, the appellation of this manuscript underwent several refinements as articulated.

Accuracy of rating scales for evaluating aphasic patients’ psychological aspects and language function: A scoping review protocol

Reviewer 1 Comments

1) *The authors often use accuracy, reliability and validity interchangeably (e.g.*

*rows 89, 95, 113, 190), but this is sometimes misleading. Also, I suggest extracting and then reporting in a result table more measurement properties (other than reliability and validity) of the researched rating scales such as sensitivity, specificity and likelihood of a positive and a negative test. Additionally, it is appropriate to report details about these aspects (e.g. for reliability: both test-retest and inter-observer reliability; for validity its different forms such as construct, content and criterion as well as internal and external validity).*

Thank you for your suggestions. We have revised the text as follows.

Line 78-81

The first purpose of this scoping review is to identify the domains of language function, communication ability, well-being, and QOL of PWA with low accuracy in rating scales based on data from the Japanese database (Ichushi web) and several international databases.

Line 83-86

Furthermore, the third purpose is to present differences in assessment scales for PWA in other countries and in Japan and to estimate the rehabilitation intervention for PWA in Japan and the accuracy of the assessment scales.

Line 195-198

Our scoping review will be significant in that it will collect data as thoroughly as possible, highlight data on the accuracy of rating scales of language function, communication ability, well-being, and QOL of PWA, and contribute to the enhancement of rehabilitation for PWA as well as the improvement of test accuracy.

Line 172-184

The presented table encompasses various essential aspects, such as the publication year, author’s name, pertinent PWA characteristics (e.g., age, gender, education level, length of time since stroke occurrence, stroke type, stroke frequency, hemisphere affected, and extent of injury), cognitive function, PWA assessor’s profession, domains assessed by the PWA rating scale, and the quality of development and demonstration of the PWA rating scale, including sample size, sampling methods, sample representativeness, and blinding. Moreover, the table includes reported accuracy, sensitivity, specificity, reliability measures (e.g., internal consistency, retest reliability, and interrater reliability), and an overview of statistical procedures related to the PWA rating scale’s validity (content validity, construct validity, and criterion-related validity). In the case of a diagnostic test to determine the presence/absence of aphasia, the table shall also present the scale used to validate the test and its cutoff values.

*2) In the inclusion criteria I suggest adding a criterion for a minimum number of patients enrolled in each study (e.g. at least 20 patients for article).*

Thank you for your suggestion. We have made the following corrections to Table 1 and additions to the text.

Table 1. Inclusion and exclusion criteria

| **Inclusion criteria** | **Exclusion criteria** |
| --- | --- |
| The literature is primarily concerned with rating scales aimed at not <20 individuals aged 18 years or older diagnosed with stroke-induced aphasia. This literature comprises observational and empirical studies, as well as research specifically aimed at developing, testing the reliability of and validating rating scales that assess various aspects of poststroke aphasic individuals' language functionality, communication abilities, overall well-being, and QOL. | Literature focusing on aphasia from factors other than stroke (head trauma, Alzheimer's disease, cerebrovascular dementia, dementia with Lewy bodies, primary progressive aphasia, frontotemporal dementia, and progressive supranuclear palsy), literature also focusing on higher brain dysfunction other than aphasia, literature focusing on aphasic under 18 years old, literature focusing on aphasia syndromes of stroke, literature focusing on training for people with aphasia after stroke, literature published before 1995, literature in languages other than English and Japanese, intervention studies, scoping reviews, systemic reviews, meta-analysis, case studies, and letter papers. |

Line 135-138

The first inclusion criterion is the exclusive incorporation of rating scales duly validated with a minimum sample size of 20 participants with PWA aged 18 years or above and supplementation by a cohort with aphasia.

*3) In Table 1 under the column “exclusion criteria” primary progressive dementia is maybe primary progressive aphasia ?*

Thank you for your careful review. We have revised Table 1 as follows

Table 1. Inclusion and exclusion criteria

| **Inclusion criteria** | **Exclusion criteria** |
| --- | --- |
| The literature is primarily concerned with rating scales aimed at not <20 individuals aged 18 years or older diagnosed with stroke-induced aphasia. This literature comprises observational and empirical studies, as well as research specifically aimed at developing, testing the reliability of and validating rating scales that assess various aspects of poststroke aphasic individuals' language functionality, communication abilities, overall well-being, and QOL. | Literature focusing on aphasia from factors other than stroke (head trauma, Alzheimer's disease, cerebrovascular dementia, dementia with Lewy bodies, primary progressive aphasia, frontotemporal dementia, and progressive supranuclear palsy), literature also focusing on higher brain dysfunction other than aphasia, literature focusing on aphasic under 18 years old, literature focusing on aphasia syndromes of stroke, literature focusing on training for people with aphasia after stroke, literature published before 1995, literature in languages other than English and Japanese, intervention studies, scoping reviews, systemic reviews, meta-analysis, case studies, and letter papers. |

*4) In the section “Stage 4: Illustrating the results” I suggest to consider to extract and report other aspects that could influence the test validity and reliability such as the stroke type (ischemic vs hemorrhagic) and its characteristics (the affected hemisphere, the extent of the lesion, the existence of a previous important vascular disease that could reduce the functional reserve). In fact, reliability and validity may change on the basis of the number and types of the brain areas involved in a stroke. Also, it is extremely important taking into account the acute, subacute or chronic setting of the selected study, or, at least, reporting in table the elapsed time from the stroke and the test administration.

5) The authors will have to provide information on methodological quality for each validation study comprehensive of the reference test used, the specialist that assessed the aphasia-related features, the used cut-off and the presence or absence of blinding.*

Thank you for your suggestion. We have added the following text in the revised manuscript.

Line 156-164

The obtained results encompass a wide array of information, including the author's name, publication year, study title, country of origin, study purpose, sampling method, sample characteristics (sex, age, educational background, presence/absence of prior stroke, and cognitive assessment results), stroke characteristics (stroke type, hemisphere affected, and extent and duration of injury), sample size, PWA assessor’s profession, rating scale utilized for PWA evaluation, rating scale domains, details of the rating scale development process, information on rating scale sensitivity and specificity, and information related to the reviews of the rating scale’s reliability and validity.

Line 172-184

The presented table encompasses various essential aspects, such as the publication year, author’s name, pertinent PWA characteristics (e.g., age, gender, education level, length of time since stroke occurrence, stroke type, stroke frequency, hemisphere affected, and extent of injury), cognitive function, PWA assessor’s profession, domains assessed by the PWA rating scale, and the quality of development and demonstration of the PWA rating scale, including sample size, sampling methods, sample representativeness, and blinding. Moreover, the table includes reported accuracy, sensitivity, specificity, reliability measures (e.g., internal consistency, retest reliability, and interrater reliability), and an overview of statistical procedures related to the PWA rating scale’s validity (content validity, construct validity, and criterion-related validity). In the case of a diagnostic test to determine the presence/absence of aphasia, the table shall also present the scale used to validate the test and its cutoff values.
